# Biomechanical Effects of a Novel Anatomic Titanium Mesh Cage for Single-Level Anterior Cervical Corpectomy and Fusion: A Finite Element Analysis

**DOI:** 10.3389/fbioe.2022.881979

**Published:** 2022-06-24

**Authors:** Ke-rui Zhang, Yi Yang, Li-tai Ma, Yue Qiu, Bei-yu Wang, Chen Ding, Yang Meng, Xin Rong, Ying Hong, Hao Liu

**Affiliations:** ^1^ Department of Orthopedic West China Hospital, Sichuan University, Chengdu, China; ^2^ Department of Applied Mechanics, Sichuan University, Chengdu, China; ^3^ Department of Operation Room, West China Hospital, Sichuan University, Chengdu, China

**Keywords:** novel anatomic titanium mesh cage, traditional titanium mesh cage, cage subsidence, implant-related complications, anterior cervical corpectomy and fusion, adjacent segment degeneration, finite element analysis

## Abstract

**Background:** The traditional titanium mesh cage (TTMC) has become common as a classical instrument for Anterior Cervical Corpectomy and Fusion (ACCF), but a series of complications such as cage subsidence, adjacent segment degeneration (ASD), and implant-related complications by using the TTMC have often been reported in the previous literature. The aim of this study was to assess whether a novel anatomic titanium mesh cage (NTMC) could improve the biomechanical condition after surgery.

**Methods:** The NTMC model consists of two spacers located on both sides of the TTMC which match the anatomic structure between the endplates by measuring patient preoperative cervical computed tomography (CT) data. The ranges of motion (ROMs) of the surgical segments and the stress peaks in the C6 superior endplates, titanium mesh cage (TMC), screw–bone interface, anterior titanium plate, and adjacent intervertebral disc were compared.

**Results:** Compared with the TTMC, the NTMC reduced the surgical segmental ROMs by 89.4% postoperatively. The C6 superior endplate stress peaks were higher in the TTMC (4.473–23.890 MPa), followed by the NTMC (1.923–5.035 MPa). The stress peaks on the TMC were higher in the TTMC (47.896–349.525 MPa), and the stress peaks on the TMC were lower in the NTMC (17.907–92.799 MPa). TTMC induced higher stress peaks in the screw–bone interface (40.0–153.2 MPa), followed by the NTMC (14.8–67.8 MPa). About the stress peaks on the anterior titanium plate, the stress of TTMC is from 16.499 to 58.432 MPa, and that of the NTMC is from 12.456 to 34.607 MPa. Moreover, the TTMC induced higher stress peaks in the C3/4 and C6/7 intervertebral disc (0.201–6.691 MPa and 0.248–4.735 MPa, respectively), followed by the NTMC (0.227–3.690 MPa and 0.174–3.521 MPa, respectively).

**Conclusion:** First, the application of the NTMC can effectively decrease the risks of TMC subsidence after surgery. Second, in the NTMC, the stresses at the anterior screw-plate, bone–screw, and TMC interface are much less than in the TTMC, which decreased the risks of instrument-related complications after surgery. Finally, increases in IDP at adjacent levels are associated with the internal stresses of adjacent discs which may lead to ASD; therefore, the NTMC can effectively decrease the risks of ASD.

## Introduction

Anterior cervical corpectomy and fusion (ACCF) is an effective treatment method for various cervical disorders, including cervical spondylosis myelopathy, ossified posterior longitudinal ligaments (OPLL), trauma, tumors, and rheumatoid arthritis.(Ji, Yu, Yan, Wang, Hou, Hou and Cai 2020, Missori, Domenicucci and Marruzzo 2018; Zeng, Duan, Yang, Wang, Hong, Lou, Ning and Liu 2018). ACCF has been widely accepted with satisfactory postoperative prognosis because of removing the direct decompression of the spinal cord and offering immediate stabilization of the surgical segments (Fengbin, Jinhao, Xinyuan, Xinwei, Yu and Deyu 2013; Yang, Chen, Liu, Song, Kong, Zeng, Xue and Ren 2013).

The use of the traditional titanium mesh cage (TTMC) has become the main method for cervical reconstruction during ACCF surgery (Fehlings, Smith, Kopjar, Arnold, Yoon, Vaccaro, Brodke, Janssen, Chapman, Sasso, Woodard, Banco, Massicotte, Dekutoski, Gokaslan, Bono and Shaffrey 2012). Although this method maintains immediate anterior column stability with good biocompatibility after surgery and has a relatively high bone fusion rate, the incidence of postoperative titanium mesh cage (TMC) subsidence reported in the previous literature is as high as 90% (Fengbin, Jinhao, Xinyuan, Xinwei, Yu and Deyu 2013; Yang, Chen, Liu, Song, Kong, Zeng, Xue and Ren 2013). TMC subsidence may be correlated with poor clinical efficacy or poor neurological recovery and can even lead to symptom recurrence, deterioration of nerve function, failure of internal fixation, and revision surgery (Mo, Li, Jia, Yang, Wong and Fan 2017; Wu, Meng, Wang, Rong, Hong, Ding, Chen and Liu 2019). Moreover, an instrument-related complication is a more serious type of complication, which includes plate fracture, broken screw, or TMC dislodgement, and may lead to a more serious set of consequences. Although there are many risk factors related to TMC subsidence and instrument-related complications, the structural improvement of the TMC is one of the most important methods to solve the problem in clinical practice.

For ACCF using the TTMC, the contact area between the TMC and endplate is limited. The upper and lower endplates are all characterized by irregular anatomical shapes. After TMC implantation, contact with the endplate through the edge of the titanium mesh and the match is poor. The contact area between the TMC and endplate is small, which is point-to-point contact. Moreover, this kind of point contact causes relatively concentrated stress, which easily leads to TMC subsidence and instrument-related complications after surgery. Therefore, it is of great importance to find a novel anatomic titanium mesh cage (NTMC) consisting of two spacers located on both sides of the TMC which match the anatomic structure between the endplates and change point-to-point contact into face-to-face contact, which can avoid uneven stress distribution and decrease the incidence of postoperative TMC subsidence and instrument-related complications.

For this purpose, an NTMC was designed, whose ends possessed enlarged spacers which match the anatomic shape between the endplates by measuring patient preoperative cervical CT data. The mechanical properties were analyzed by using a three-dimensional finite element (FE) analysis to analyze whether this NTMC could effectively improve the biomechanical performance in ACCF.

## Materials and Methods

### Finite Element Model of the Intact Lower Cervical Spine

An intact C2-7 FE model was constructed with the following steps. Computed tomography (CT) images (SOMATOM Definition AS+, Siemens, Germany) of the C2-7 cervical spine were obtained from a young healthy volunteer (37 years of age; height, 172 cm; weight, 65 kg) and were then imported into Mimics 17.01 (Materialise Corporation, Belgium) to reconstruct the surface model of each vertebra. Solid models of the cortical shell, cancellous bone, and intervertebral disk were constructed in Geomagic Studio 2015 (Raindrop Geomagio Inc. United States). Meshes of the bones, intervertebral discs, and ligaments were constructed using Hypermesh 14.0 (Atair Corporation. United States) and imported into Abaqus 6.14 (Dassault System. France) for material property definitions, model assembly, and FE analysis (Liu, Lu, Wang, Sun, Li and He 2019).

**FIGURE 1:**
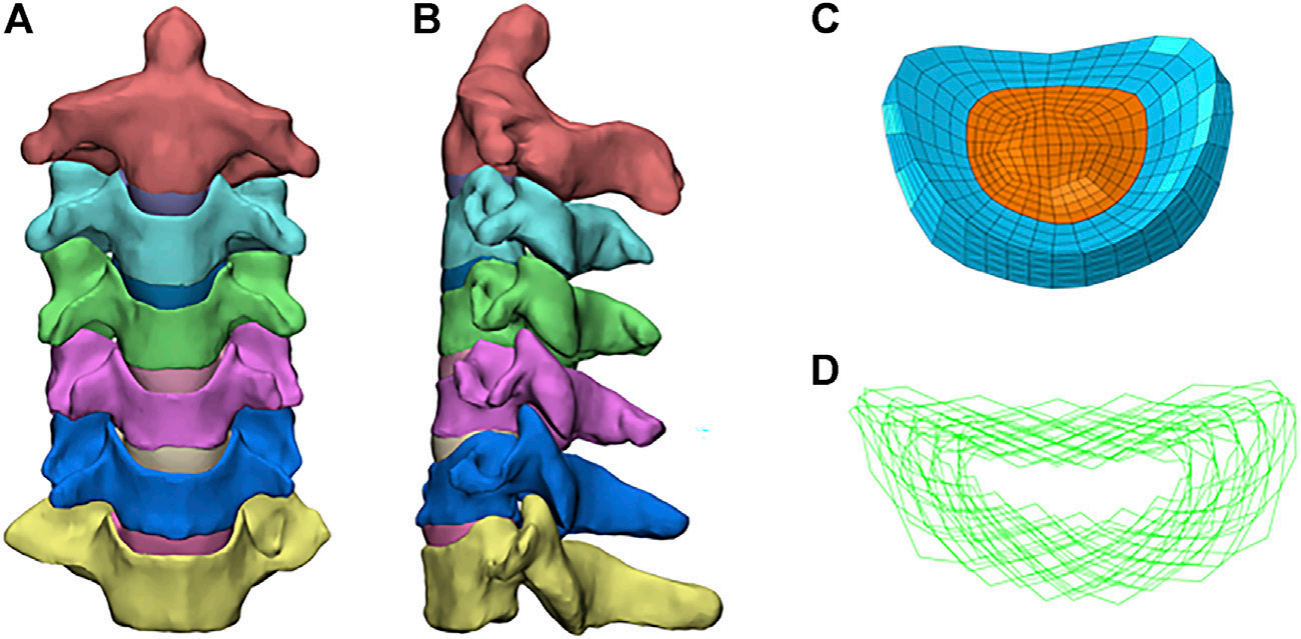
Finite element model of the intact C2–C7 cervical spine: **(A)** front view; **(B)** sagittal view; **(C)** annulus fiber; **(D)** intact intervertebral disk.

Figure 1 shows the FE model of the intact C2-7 cervical spine, which consisted of six vertebrae, five intervertebral disks, the anterior longitudinal ligament, the posterior longitudinal ligament, the capsular ligament, the interspinous ligament, the supraspinous ligament, and the ligamentum flavum. A 0.5-mm-thick shell consisting of cortical bone (Denoziere and Ku 2006) and the nucleus pulposus was modeled as an incompressible inviscid fluid, and the intervertebral disc was divided into the annulus fibrosus and nucleus pulposus, with a volume ratio of 7:3 (Kallemeyn, Gandhi, Kode, Shivanna, Smucker and Grosland 2010). The annulus fibrosus was modeled as an annulus ground substance embedded with annulus fibers. Annulus fibers surrounded the ground substance with an inclination to the transverse plane between 15° and 45° (Mo, Li, Jia, Yang, Wong and Fan 2017). Hypoelastic material properties were assigned to the ligament according to the stress–strain curves that were published previously (Kallemeyn, Gandhi, Kode, Shivanna, Smucker and Grosland 2010). A convergence analysis was performed to ensure that the maximum changes in the strain energy were <5% (Ayturk and Puttlitz 2011; Jones and Wilcox 2008). The element types and material properties used in the FE model are shown in Table 1, which is based on previous publications (Kallemeyn, Gandhi, Kode, Shivanna, Smucker and Grosland 2010; Wu, Meng, Wang, Rong, Hong, Ding, Chen and Liu 2019).

**TABLE 1:** Material properties assigned to the finite element model.

component	Element type	Young’s modulus (MPa)	Poisson ratio	Cross-sectional area (mm^2^)
Cortical bone	C3D4	12,000	0.29	
Cancellous bone	C3D4	450	0.29	
Nucleus pulpous	C3D8H	1	0.49	
Anterior longitudinal	T3D2	30	0.3	6.1
Posterior longitudinal	T3D2	20	0.3	5.4
Capsular	T3D2	20	0.3	46.6
Ligamentum flavum	T3D2	1.5	0.3	5.0
Interspinous	T3D2	1.5	0.3	13.1
Supraspinous ligament	T3D2	1.5	0.3	5.0
Facet joint cartilage	C3D4	10.4	0.4	
Titanium alloy	C3D4	110,000	0.3	
Screws	C3D4	110,000	0.3	
Cages	C3D4	110,000	0.3	
Spacers	C3D4	110,000	0.3	
Endplates	C3D4	5.6	0.3	
Annulus fibers	T3D2	110	0.3	

PEEK, polyetheretherketone.

### FE Model of the ACCF Procedures

**FIGURE 2:**
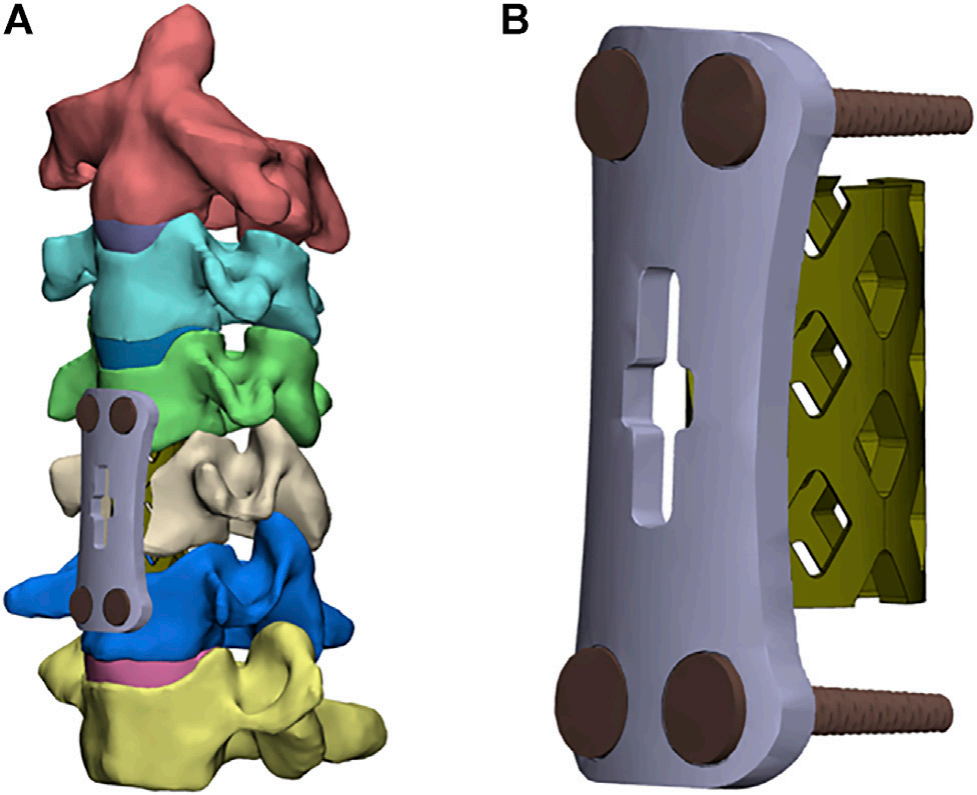
Finite element models of the surgical procedures: **(A)** front view of ACCF using the TTMC; **(B)** the structure of the TTMC.

Figures 2A,B show the FE models of ACCF at C4-6. The corpectomy of C5 was simulated by removing the C4-5 and C5-6 intervertebral disks; two-thirds of the vertebral body in C5; and the corresponding anterior and posterior longitudinal ligaments (Liu, Lu, Wang, Sun, Li and He 2019). After the corpectomies, a TTMC (Johnson & Johnson, United States) with a 12-mm diameter was trimmed into a suitable length and implanted into the intervertebral space. Both ends of the TTMC were ensured to be in close contact with the corresponding endplates. The contact area in the cage-endplate interface end was 0.27 cm^2^. An anterior plate-screw system was placed at C4-6 to further stabilize the surgical segment. The length and width of the anterior titanium plate were 36 and 12 mm, respectively, and the length and diameter of the screws were 12 and 3 mm, respectively (TTMC internal fixation system).

**FIGURE 3:**
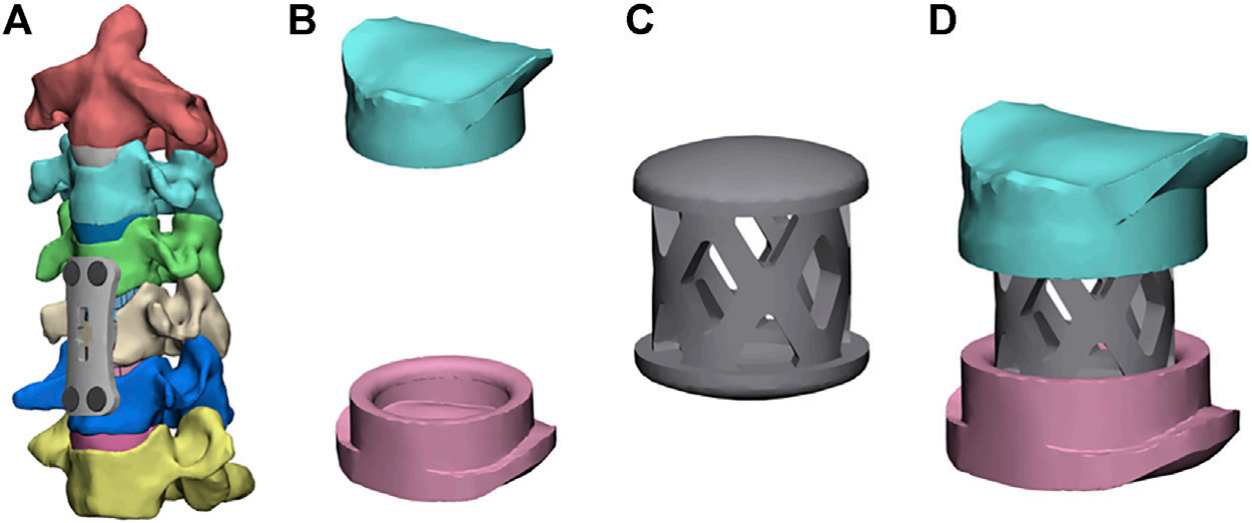
Finite element models of the surgical procedures: **(A)** front view of ACCF using the NTMC, **(B)** the structure of spacers, **(C)** the structure of the TMC, and **(D)** TMC and the spacer are connected by the slot structure.

Figures 3A,B show the FE model of ACCF using the NTMC for interbody fusion. After the corpectomies, an NTMC with a 12-mm diameter was implanted into the space. Both ends of the NTMC were enlarged by adding a spacer to each end, which matches the shapes between the endplates by measuring the patient preoperative CT scan data, and the TMC was fixed to the spacer by the slot structure (Figure 3C). Satisfactory matching between the endplate and spacer was achieved using the Boolean calculation to remove the portion that overlapped with the vertebral body. The contact area in the spacer–endplate interface was 3.63 cm^2^. For all surgical models, the interfaces at the cage endplate and screw bone were defined as a tied contact condition to simulate a complete fusion status (Zhao, Chen, Liu, Elsamaloty, Liu, Li, Elgafy, Zhang and Wang 2018).

### Loading and Boundary Conditions

The FE model of intact C2−C7 segments was fixed at the inferior endplate of C7. Follower loads of 75 N were used to simulate muscle force and head weight. A 1.0-Nm moment and a 75-N follower load were applied to the odontoid of the C2 vertebrae to produce flexion, extension, lateral bending, and axial rotation (Wu, Meng, Liu, Wang, Hong, Rong, Ding and Chen 2019). The surgical segment ranges of motion (ROMs), the stress of the C6 superior endplates, TMC, screw–bone interface, anterior titanium plate, and adjacent intervertebral disc were compared between the constructs of ACCF using the TTMC and ACCF using the NTMC. Based on previous studies and literature data, C4/5 and C5/6 were chosen as the implanted levels because they are the most frequently involved levels in clinical practice (Ouyang, Lu, He, Gao, Cai and Jin 2019).

## Results

### Model Validation

**FIGURE 4:**
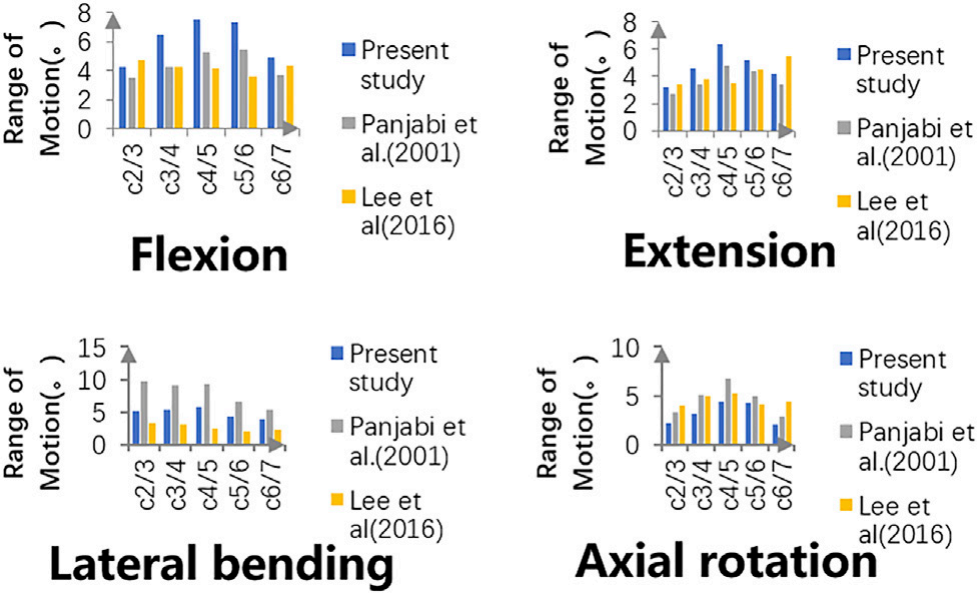
Intersegmental ROMs of the intact model are compared with those of previously published studies, and validity has been proven.

The intersegmental ROMs at C2-3, C3-4, C4-5, C5-6, and C6-7 were 4.29°, 6.49°, 7.45°, 7.35°, and 4.89°, respectively, in flexion; 3.16°, 4.57°, 6.32°, 5.22°, and 4.21°, respectively, in extension; 5.14°, 5.42°, 5.67°, 4.21°, and 3.85°, respectively, in bending; and 2.14°, 3.15°, 4.36°, 3.60°, and 2.08°, respectively, in rotation. As shown in Figure 4, the intersegmental ROMs in each motor direction showed good agreement with the outcomes of previous publications, where the consistency can be as high from 61.3 to 95.1% (Panjabi, Crisco, Vasavada, Oda, Cholewicki, Nibu and Shin 2001; Lee, Park, Kim and Jahng 2016). Moreover, in the study of cadaver specimens *in vitro*, the intersegmental ROMs at C2-3, C3-4, C4-5, C5-6, and C6-7 were 3.90°, 7.05°, 6.02°, 7.93°, and 5.33°, respectively, in flexion; 2.21°, 4.97°, 5.32°, 7.81°, and 5.61°, respectively, in extension; 4.32°, 6.54°, 4.07°, 4.28°, and 2.79°, respectively, in bending; and 2.37°, 3.97°, 5.13°, 6.22°, and 3.63°, respectively, in rotation, where the consistency is up to 98.4% and can prove the validity of the model to a certain extent (Kallemeyn, Gandhi, Kode, Shivanna, Smucker and Grosland 2010).

### ROMs of the Surgical Segments

**FIGURE 5:**
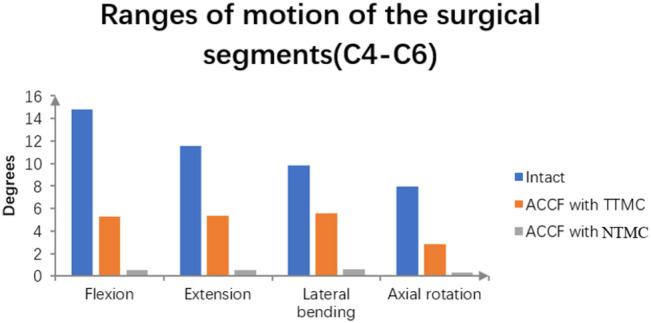
Using the intact model as a reference, comparisons of the ROMs of the surgical segments (C4–C6) between the TTMC and NTMC models has been performed.

As shown in Figure 5, the ROMs of the intact C4-6 model in flexion, extension, bending, and rotation were 14.8°, 11.55°, 9.86°, and 7.96°, respectively. Postoperatively, the surgical segment ROMs of ACCF using a TTMC and ACCF using the NTMC were 5.28° and 0.56°, respectively, in flexion; 5.38° and 0.52°, respectively, in extension; 5.55° and 0.59°, respectively, in bending; and 2.85° and 0.31°, respectively, in rotation. The differences in the surgical segment ROMs between the TTMC and NTMC can be as much as 90.3%, and the differences were significant between the two groups, which is similar to previous literature (La Barbera, Larson, Rawlinson and Aubin 2021).

### Cortical Endplate Stresses

**FIGURE 6:**
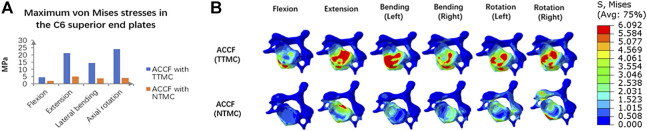
**(A)** Comparisons of the maximum von Mises stresses in the C6 superior endplate between the TTMC and NTMC models; **(B)** Stress cloud map of the C6 superior endplates between TTMC and NTMC models.

Figure 6A shows the maximum stresses in the C6 superior endplates. The endplate stress peaks were higher in the construct of ACCF using a TTMC, wherein the stresses were 4.47, 21.27, 14.35, and 23.89 MPa in flexion, extension, bending, and rotation, respectively. In the same direction of movement, the endplate stress peaks were lower by using the NTMC in ACCF, which reduced to 1.92, 5.04, 3.81, and 3.97 MPa, respectively. The stress distributions in the C6 superior endplates are shown in Figure 6B.

### Stress at the TMC

**FIGURE 7:**
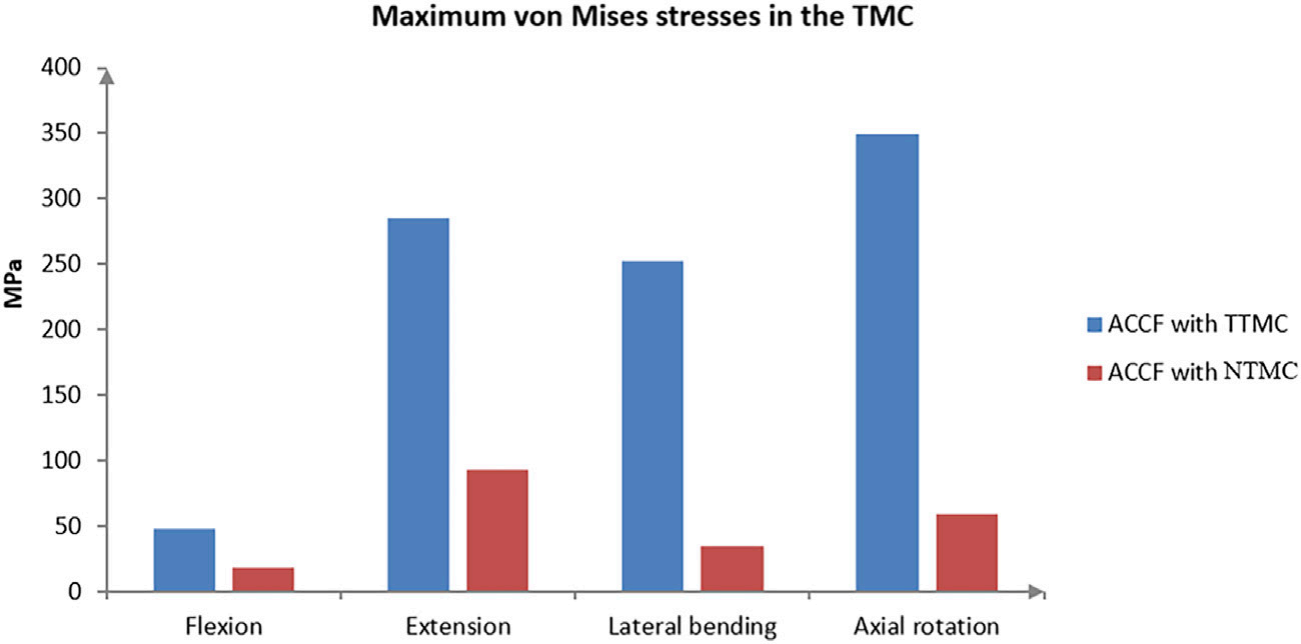
Comparisons of the maximum von Mises stresses in the TMC between TTMC and NTMC models.

The maximum von Mises stresses in the TMC are shown in Figure 7. In the ACCF using a TTMC model, the stresses at the TMC interface in flexion, extension, lateral bending, and axial rotation were 47.90, 285.45, 252.04, and 349.53 MPa, respectively. In the ACCF using an NTMC model, the stresses at the TMC interfacial in flexion, extension, lateral bending, and axial rotation were 17.91, 92.80, 34.73, and 59.01 MPa, respectively.

### Stress at the Bone–Screw Interface

**FIGURE 8:**
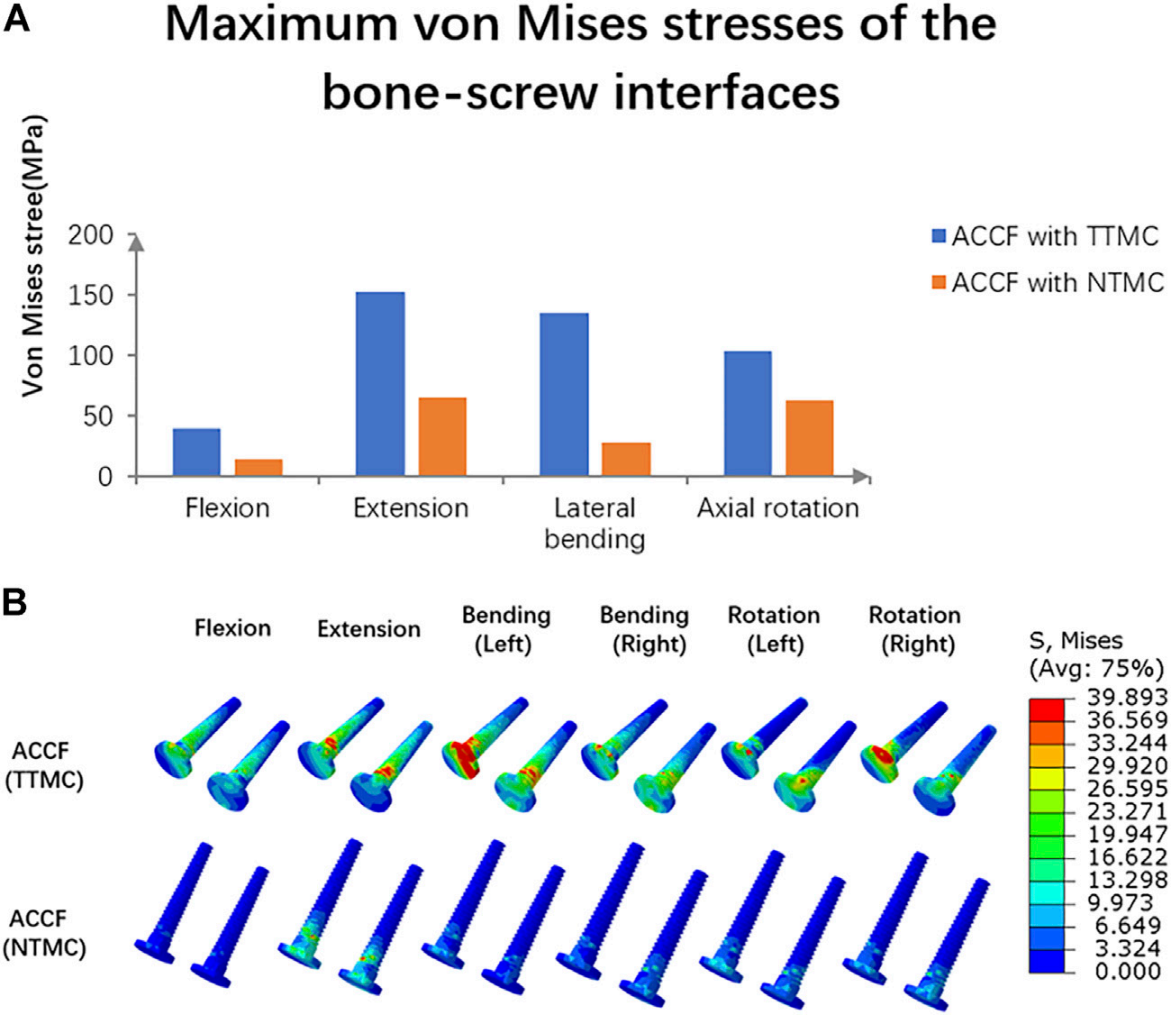
**(A)** Comparisons of the maximum von Mises stresses in the C6 screw–bone interface between TTMC and NTMC models; **(B)** Stress cloud map of the C6 screw–bone interface between TTMC and NTMC models.

The maximum von Mises stresses in the C6 screw interface are shown in Figure 8A. In the ACCF using a TTMC model, the stresses at the bone–screw interface in flexion, extension, lateral bending, and axial rotation were 40.04, 153.18, 134.83, and 103.57 MPa, respectively. In the ACCF using an NTMC model, the stresses at the bone-screw interface in flexion, extension, lateral bending, and axial rotation were 14.82, 65.49, 28.17, and 63.44 MPa, respectively. The stress cloud map in the C6 screw–bone interfacial stresses between two models is shown in Figure 8B.

### Fixation Systems Stresses

**FIGURE 9:**
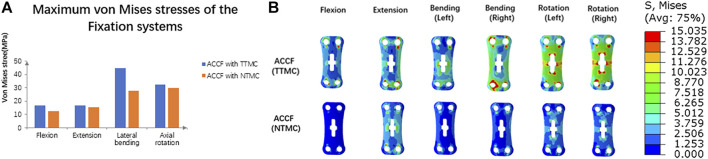
**(A)** Comparisons of the maximum von Mises stresses of the fixation systems between TTMC and NTMC models; **(B)** Stress cloud map of the anterior titanium plate between TTMC and NTMC models.

The maximum von Mises stresses of the fixation systems are shown in Figure 9. In the ACCF using a TTMC model, the stresses at the anterior titanium plate in flexion, extension, lateral bending, and axial rotation were 16.51, 16.47, 44.49, and 32.54 MPa, respectively. In the ACCF using an NTMC model, the stresses at the anterior titanium plate in flexion, extension, lateral bending, and axial rotation were 12.48, 15.17, 27.93, and 30.02 MPa, respectively.

### Stress on the C3/4 Intervertebral Disc

**FIGURE 10:**
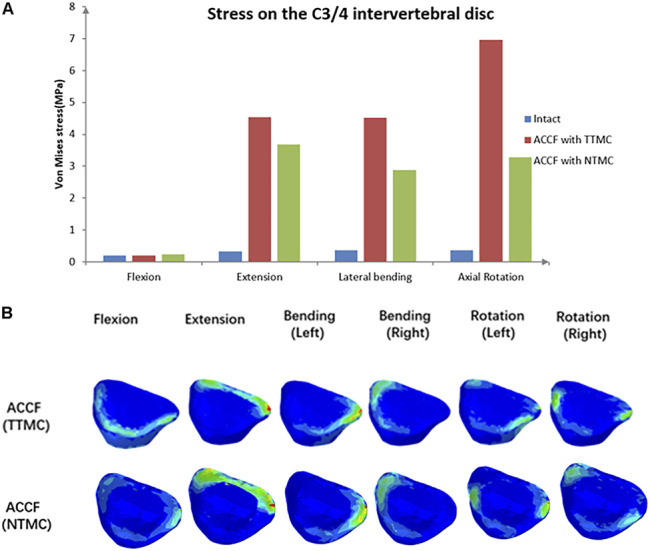
**(A)** Comparisons of the maximum von Mises stresses in the IDP of the C3/4 segment among the intact model, TTMC model and NTMC model; **(B)** Stress cloud map of IDP of the C3/4 segment between TTMC and NTMC models.

Intradiscal pressure (IDP) measures at the supra-adjacent (C3/4) segment are presented in Figure 10. Compared with the TTMC model, the stress on the upper (C3/4) adjacent intervertebral disc in the NTMC model was lower during flexion, extension, lateral bending, and rotation. In the TTMC model, the maximum von Mises stresses on the C3/4 intervertebral disc were 0.20 MPa during flexion; 4.55 MPa during extension; 4.53 MPa during lateral bending; and 6.96 MPa during rotation. In the NTMC model, the maximum von Mises stresses on the C3/4 intervertebral disc were 0.23 MPa during flexion; 3.69 MPa during extension; 2.87 MPa during lateral bending; and 3.27 MPa during rotation; and in the intact model, the maximum von Mises stresses on the C3/4 intervertebral disc were 0.19 MPa during flexion; 0.32 MPa during extension; 0.37 MPa during lateral bending; and 0.36 MPa during rotation. The stress distributions on the C3/4 intervertebral disc are shown in Figure 10B.

### Stress on the C6/7 Intervertebral Disc

**FIGURE 11:**
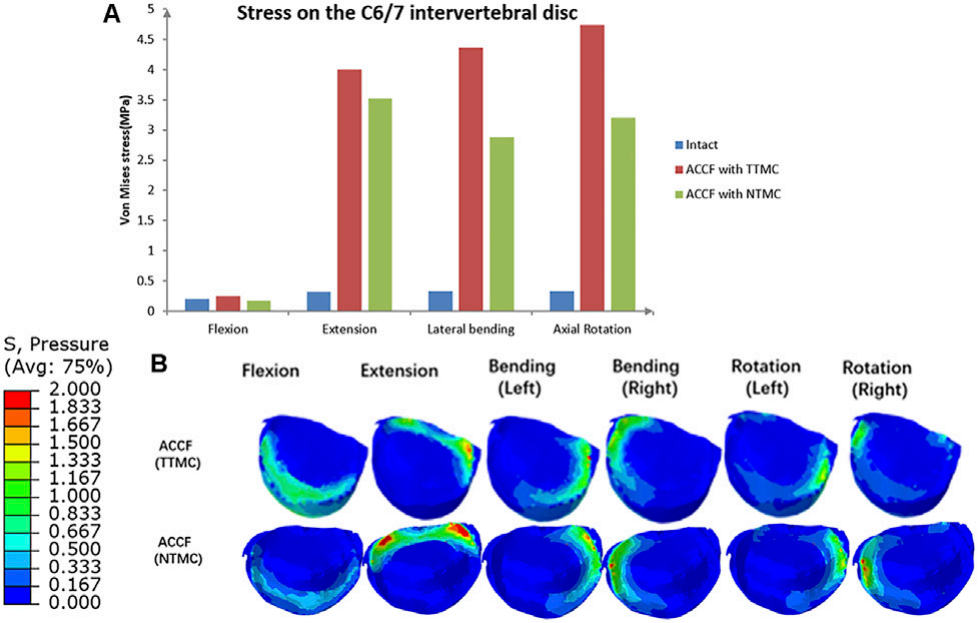
**(A)** Comparisons of the maximum von Mises stresses in the IDP of the C6/7 segment among the intact model, TTMC model, and NTMC model; **(B)** Stress cloud map of IDP of the C6/7 segment between TTMC and NTMC models.

IDP measures at the infra-adjacent (C6/7) segment are presented in Figure 11. In these two models, the NTMC group had lower stress on the intervertebral disc than the TTMC group. In the TTMC group, the maximum von Mises stresses on the C6/7 intervertebral disc were 0.25 MPa during flexion; 4.00 MPa during extension; 4.37 MPa during lateral bending; and 4.74 MPa during rotation. In the NTMC group, the maximum von Mises stresses on the C6/7 intervertebral disc were 0.17 MPa during flexion; 3.52 MPa during extension; 2.88 MPa during lateral bending; and 3.20 MPa during rotation, and in the intact model, the maximum von Mises stresses on the C6/7 intervertebral disc were 0.21 MPa during flexion; 0.32 MPa during extension; 0.33 MPa during lateral bending; and 0.33 MPa during rotation. The stress distributions on the C6/7 intervertebral disc are shown in Figure 11B.

## Discussion

### Construct Stability

This study comprehensively compared the biomechanical stabilities provided by ACCF with the TTMC model and NTMC model. As shown in the results, both models could significantly reduce the ROMs in the surgical segments compared with the intact model. Therefore, the ACCF with the TTMC model and NTMC model both can achieve strong construct stability in the surgical segments.

According to the result of the ROMs of the surgical segments, compared with the TTMC model, the ACCF with the NTMC model has smaller ROMs in the surgical segments, which means that the NTMC model has better stability. In the present study, similar to previous studies, the boundary conditions of the spacer–bone interfaces were assigned to be tied contacts to simulate the status of bony fusion (Wu, Meng, Wang, Rong, Hong, Ding, Chen and Liu 2019). Compared with the cage–bone interfaces in the TTMC model, the spacer–bone interfaces have a larger area in contact with the whole endplate, which is more beneficial to increasing anterior column stability immediately after surgery and bone fusion during the process of follow-up. As the bony fusion at the endplate space, the stiffness of the anterior column increases, which further improves the stability of the construct. A recent study found that compared with immediately after surgery, the ROMs of the surgical level were further reduced by 11.5% when bony fusion was achieved at the intervertebral space (Li, Wu, Chu, Liu, Hou, Yu and Hou 2018). In general, the NTMC model can not only improve the stability immediately after surgery but also maintain long-term stability after the operation.

### Subsidence Resistance

TMC subsidence is one of the common postoperative complications in ACCF (Wen, Lu, Wang, Liang, Gao and He 2018). The relatively high endplate interfacial stress concentration is an important factor that facilitates the cage penetrating into the endplate and inducing cage subsidence (Lu, Liang, Liu, Guo, Zhang, Yang and He 2017; Wen, Lu, Wang, Liang, Gao and He 2018). In some literature, bony endplate strain/stress threshold is also reported (Ottardi, La Barbera, Pietrogrande and Villa 2016; La Barbera, Cianfoni, Ferrari, Distefano, Bonaldi and Villa 2019; La Barbera, Cianfoni, Ferrari, Distefano, Bonaldi and Villa 2019).

Although cage subsidence has little influence on the clinical outcomes in some patients, some severe cases may induce kyphosis, neurologic deterioration, and instrumental-related complications because of the significant decrease in intervertebral height and the subsequent increase in stress load within the anterior titanium plate (Takase, Murata, Sato, Tanaka, Miyazaki, Yoshizumi, Tateishi, Kawahara and Yamamoto 2018).

To address these issues, the NTMC model consisting of two spacers located on both sides of the TMC that match the shapes of the upper and lower endplate was developed, where the contact area in the spacer–endplate interface was 3.57 cm^2^. The outcomes of endplate stresses showed that ACCF using the TTMC for vertebral body construction induced approximately 3 to 5-fold greater stress peaks on the C6 endplate than the NTMC model (4.5–23.9 vs. 1.9–5.0 MPa, respectively). For the TTMC model, the contact area between the TMC end and the endplate is small (the contact area was 0.31 cm^2^), which results in a large stress concentration and leads to cage subsidence.

The present study concluded that the larger the spacer–endplate interface contact area, the lower the subsidence rate of the TMC. In addition, some new cages also have been reported to support this viewpoint. These new cages offered larger contact areas with the endplate by enlarging the surface area and simulating the endplate shapes in the cage end to prevent excessive stress concentration, which effectively dispersed the stress distribution and reduced the subsidence rate (Fengbin, Jinhao, Xinyuan, Xinwei, Yu and Deyu 2013; Shamji, Zhang, Quan, Zhao, Luo, Tang, Li, Zhou and Jiang 2014). Due to the anatomical structure of the cervical spine among patients being different, it is difficult to achieve perfect anatomical matching with the endplates by using new cages (Lou, Liu, Rong, Li, Wang and Gong 2016). However, the postoperative results found that these new cages are still in close contact with the endplates, effectively increasing the contact area, rebuilding the intervertebral height, and dispersing the endplate stress. By simulating the shape of the endplate, these new cages significantly decreased the interval between the spacer and the endplate compared with the conventional TMC with a flat end (Fengbin, Jinhao, Xinyuan, Xinwei, Yu and Deyu 2013; Shamji, Zhang, Quan, Zhao, Luo, Tang, Li, Zhou and Jiang 2014). Therefore, the reduction of the interval leads to a significant increase in the contact area, which further reduces the stress concentration and decreases the risk of postoperative subsidence (Ordway, Rim, Tan, Hickman and Fayyazi 2012).

It can be seen from the endplate stress nephograms that due to the limited interface contact area between the TMC and endplate, the stress distribution of using the TTMC in ACCF is mainly concentrated on the anterior and lateral parts of the endplate. By using the NTMC in ACCF, due to the spacer simulated the shape of the endplate, the contact area at the cage endplate significantly expanded, and the stress distribution on the endplate became homogeneous, which reduced the concentration of stress and decreased the subsidence rate of the TMC.

### Risks of Instrument-Related Complications

Instrument-related complications include plate fracture, broken screw, or TMC dislodgement, which can lead to risks such as neck pain, compression of the esophagus, compression of the spinal cord, and even paralysis after surgery, and most of them even require revisions. Therefore, the NTMC model was designed to decrease the risks of instrument-related complications.

A previous study found that the lowest maximum load to identify an endplate failure event by using the conventional TMC is about 1300 N, and the contact area between the endplate and the TMC is about 30.5 mm^2^. From this, we can conclude that the stress threshold of the endplate is about 42.6 Mpa. For another, the lowest maximum load to identify an endplate failure event by using the novel TMC is about 2100 N (Lu, Liang, Liu, Guo, Zhang, Yang and He 2017). However, the present methods for a reasonable estimation of the stress threshold for subsidence are limited, and because the physiological curvature of the cervical spine is lordotic, the load on the anterior spine would be eccentric with respect to the posterior spine. Therefore, any compressive load is not only pure axial translation but also involves a rotation. As mentioned above, since the spine rotation is not taken into account, our previous stress applied at the interface between the cage and the endplate cannot lead to a reasonable estimation of the stress threshold for subsidence. In addition, due to the subsidence failure being also associated with huge shear loads and bending moments, even if the load was following the spine curvature (follower load), we would not be sure that the load would be uniformly distributed across the cage–endplate interface. Accordingly, we would make relevant targeted optimizations in future research.

Compared with the ACCF using a TTMC model, the stresses at the anterior screw–plate interface, bone–screw interface, and TMC in the NTMC model are much less. According to the load-shared results, we found that the NTMC model can effectively reduce the stress load on the TMC. The reasons for the lower stress loads at the anterior screw–plate interface, bone–screw interface, and TMC by implanting the NTMC were attributed to the increase in the contact area at the spacer–endplate interface and the dispersion of the stress distribution, which offered greater stability for the anterior column (Fengbin, Jinhao, Xinyuan, Xinwei, Yu and Deyu 2013; Lu, Liang, Liu, Guo, Zhang, Yang and He 2017) and decreased the risks of instrument-related complications in ACCF.

### Risks of the Degeneration at Adjacent Discs

It is of great importance to evaluate the changes in internal stresses at adjacent levels of surgical segments by measuring the IDP (Barrey, Campana, Persohn, Perrin and Skalli 2012). Increases in IDP at adjacent levels may lead to adjacent segment degeneration (ASD), which affected patient postoperative recovery and quality of life (Zeng, Duan, Yang, Wang, Hong, Lou, Ning and Liu 2018). Increases in IDP at adjacent levels after surgery may be relevant to many reasons, such as discogenic pathology, changes in cervical curvature, and subsequent pain (Eck, Humphreys, Lim, Jeong, Kim, Hodges and An 2002). The present study found that the insufficient stability of the anterior column can also lead to increases in IDP. In addition, the stress load led to the intervertebral disc cells being stimulated by stresses such as compressive stress and tensile stress, which not only increased the change of ROM but also damaged the intervertebral disc to a large extent. Because of offering a better fixation method to improve the stabilities of the cervical spine, the adjacent IDP in the NTMC model was less than that in the TTMC model in all directions, which agreed with the changes of ROM, suggesting that the new model had the ability to delay the degeneration at adjacent discs. It can be seen from the IDP nephograms that the stress distribution of using the TTMC in ACCF is consistent with the NTMC model, and both are aligned with the direction of motion. This indicates that the stress distribution of the adjacent intervertebral disc is related to the motion direction of the cervical spine.

Moreover, the paravertebral muscle strength played a crucial role in regulating IDP. Therefore, for daily activities, patients should pay attention to the muscle strength of their neck through exercise to decrease the IDP at adjacent levels after surgery.

### Limitations

FE analysis is a traditional style for judging the diagnosis after different surgical strategies and offering treatment options. However, there are still some limitations of the current study. First, we idealized some situations within an acceptable range. The frictionless contact in the facet joint surfaces may lead to potential errors (Panzer and Cronin 2009; Li, Fogel, Liao, Tyagi and Liu 2018). Any possible micromotion among the TMC-spacers, bone-implant, and screw–bone interface was ignored, which were modeled as a tie. Second, although we operated on an *in vitro* model for surgical simulation and inserted a device in the surgical models, simplified *in vitro* models may not simulate the actual biomechanical environment during the process of surgery, especially for endplates and ligaments at the surgical segments. Third, in this research, we performed the finite element model analysis based on CT data from a 37-year-old young healthy man, which might not take the impact of degenerative pathology into account on the biomechanical properties of the spine. Finally, various types of NTMCs have different structural and biomechanical features, and the results of the current study have certain limitations and may not be applicable to other devices. Thus, the FE model may not be the best representation of the real state, and the main purpose of this research is to provide a trend rather than actual data.

## Conclusion

First, the application of the NTMC that possessed two enlarged spacers and matched the anatomic structure between the endplates seems to suggest a decreased risk of TMC subsidence in ACCF by dispersing stress, which can be proved by the stress cloud map. Second, in the ACCF with the NTMC, the stresses at the anterior screw–plate interface, bone–screw interface, and TMC are much less than those at the TTMC, which might decrease the risks of instrument-related complications after surgery and enhance the speed of postoperative bone healing so as to improve the prognosis of patients. Finally, increases in IDP at adjacent levels are associated with the internal stresses of adjacent discs which may lead to ASD; therefore, the NTMC has the potential to decrease the risks of ASD.

## Data Availability

The original contributions presented in the study are included in the article/Supplementary Material; further inquiries can be directed to the corresponding author.

## Abbreviations

ACCF, anterior cervical corpectomy and fusion; ASD, adjacent segment degeneration; CT, computed tomography; FE, finite element; IDP, intradiscal pressure; NTMC, novel anatomic titanium mesh cage; ROM, range of motion; TTMC, traditional titanium mesh cage; TMC, titanium mesh cage.
